# Genetic control of seed iron and zinc concentration in Rwandan common bean population revealed by the Genome Wide Association Study (GWAS)

**DOI:** 10.1270/jsbbs.24087

**Published:** 2025-06-18

**Authors:** Floride Mukamuhirwa, Kenta Shirasawa, Ken Naito, Edouard Rurangwa, Viateur Ndayizeye, Alphonse Nyombayire, Jean Pierre Muhire, Mahalingam Govindaraj, Norikuni Ohtake, Keiichi Okazaki, Moeko Okada, Eigo Fukai

**Affiliations:** 1 Graduate School of Science and Technology, Niigata University, 8050 Ikarashi 2-no-cho, Nishi-ku, Niigata 950-2181, Japan; 2 Department of Crop Innovation and Technology Transfer, Rwanda Agriculture and Animal Resources Development Board (RAB), P. O. Box 5016 Huye, Rwanda; 3 Kazusa DNA Research Institute, 2-6-7 Kazusa-Kamatari, Kisarazu, Chiba 292-0818, Japan; 4 Research Center of Genetic Resources, National Agriculture and Food Research Organization, 2-1-2 Kannondai, Tsukuba, Ibaraki 305-8602, Japan; 5 HarvestPlus Program, Alliance of Bioversity International and International Center for Tropical Agriculture (CIAT), Patancheru-502 324, Telangana, India

**Keywords:** common bean, GWAS, seed minerals, biofortification, Rwanda

## Abstract

Common bean (*Phaseolus vulgaris* L.) is one of the most abundantly consumed legume crops as foods worldwide. In many African countries, this crop is an important staple food because of its rich nutrients. The Great Lakes region of Central Africa, which includes Rwanda, the nation with the highest per capita consumption of common beans worldwide, is known to be a center of common bean diversity in Africa. Increasing the amount of iron and zinc in common bean for biofortification has been a key breeding goal in Rwanda and other countries. In this study, using 192 accessions, including local landraces from Rwanda, breeding materials, released varieties, and others, we performed genome wide association studies (GWAS) to determine the loci governing those traits in addition to other agronomic traits. We identified a locus that was strongly associated with seed zinc concentration and candidate genes. The information might be a great help for marker-assisted breeding of this trait in common bean.

## Introduction

Common bean (*Phaseolus vulgaris* L.) is one of the most produced and consumed legume crops worldwide for dietary purposes. Today, common bean is an extremely important crop in Africa as well as in South America where it originated. In fact, the citizens of Rwanda and Burundi, two neighboring countries in the Great Lakes region of Central Africa, consume 31.4 kg and 27.4 kg of beans per capita per year, respectively, the highest in the world. They are followed by Latin American countries and the people of other countries in the African Great Lakes region, namely Tanzania, Uganda, and Kenya (FAOATAT, https://www.fao.org/faostat/en/#data/FBS, average value of kg/cap/year from 2018 to 2022).

It is known that this crop had experienced two times of domestications (Bitocchi *et al.* 2012, Rendón-Anaya *et al.* 2017, Schmutz *et al.* 2014). The first cultivation of common bean which occurred in the middle America generated a gene pool known as Mesoamerican, and the second one occurred in the southern America resulted a gene-pool called Andean. Phylogenic relationships between the two gene-pools revealed by the genome-wide analysis of the DNA polymorphisms suggested that Andean was originated from the migrated small populations of Mesoamerican (Bitocchi *et al.* 2012, Cortinovis *et al.* 2024, Rendón-Anaya *et al.* 2017, Schmutz *et al.* 2014). It is assumed that common bean was introduced to Africa by Portuguese traders in the early 16th century. The crop is thought to be first brought to Sofala, Zanzibar and Mombasa, the major port cities on the east coast of Africa, then spread into the inner regions of the continent, and reached at the Great Lakes region of Central Africa (Greenway 1945). Today, this region is known as the center of common bean cultivation in Africa and is also considered as the second center of bean diversity, as a great diversity in their traits was already observed in the African Great Lakes region in early 20th (Trutmann 1996).

Common bean has been central to the country’s agricultural research since the 1980s, when the Rwanda Agricultural Research Institute (ISAR) was established. Farmer participatory breeding methods have furthered Rwanda’s breeding programs with an emphasis on market demand and gender considerations given the high proportion of women in the farming workforce in many parts of the country. Common bean can be grouped into two groups according to their plant structures, climbers, which are vines and need to be supported, and bushes, which are self-standing and can grow without support (Katungi *et al.* 2019). Addition to that, the middle between them is often called as semi-climbers. The breeding programs have focused on both bush and climbers. In the 2010s and 2020s, through Rwanda Agriculture and Animal Resources Development Board (RAB), newly developed bean varieties were released to farmers with success in adoption and utilization (Vaiknoras *et al.* 2019). Usually, climbers require longer time to harvest compared to bush, instead, the yield of the former is believed to be higher than the latter. In fact, it is estimated that farmers in Rwanda who switched from bush to climbers increased their yields by 23% (Katungi *et al.* 2019). In Rwanda, common bean is mostly grown in two seasons: season A, which runs from September to February of the following year; season B, which runs from March to June. Most farmers produce beans in both seasons, with a larger area in season A compared to seasons B.

Common beans are an excellent source of basic nutrients such as protein, fats, fatty acids, and carbohydrates, as well as minerals such as zinc (Zn) and iron (Fe) (Beebe *et al.* 2000). However, despite the high consumption of common bean in Rwanda, there are still some degree of anaemia among women of reproductive age (15 to 49 years old: 10% and 18% for non-pregnant and pregnant women, respectively) and children between 6 to 59 months (22%) (National Institute of Statistics of Rwanda 2021). Since micronutrients deficiencies, including Fe and Zn, are a common problem in many African countries as well as in other developing countries that consume large amounts of common beans, beside very high and wider Fe and Zn genetic variation found, this crop is a suitable target for biofortification to contribute to this problem eradication. As increasing seed Fe and Zn concentration is an important breeding target for common bean, attempts to identify the loci regulating these traits have been intensively conducted by many groups using plant populations of different regional origin, using both QTL mapping (Izquierdo *et al.* 2018) and Genome Wide Association Studies (GWAS) (Caproni *et al.* 2020, Delfini *et al.* 2021, Gelaw *et al.* 2023, Gunjača *et al.* 2021, Izquierdo *et al.* 2024, Keller *et al.* 2022, Nazir *et al.* 2022). Although many loci have been detected so far, those commonly identified between different studies were limited. In other words, the location of the loci that regulate the seed Fe and Zn concentrations depends largely on the genetic structure of the population and the environment in which they are grown. Therefore, in order to immediately use the obtained knowledge for breeding, it is important to use the possible breeding target population and perform the studies based on the phenotypes observed under the local growing environment.

In this study, to identify loci controlling Fe and Zn concentration in Rwandan common bean, we conducted GWAS using 192 accessions including landraces owned by Rwanda Agriculture and Animal Resources Development Board (RAB). In this GWAS, other three agronomic traits important for the common bean breeding were also investigated. The genome-wide genotype information obtained for GWAS was used to consider the possible constraints in genetic exchanges during the breeding programs conducted in the past.

## Materials and Methods

### Genetic material

Supplemental Table 1 shows the list of the accessions. The population used in this study was composed of one hundred ninety-two (192) accessions, including 23 landraces and 133 improved (released varieties and breeding lines) from Rwanda, and 36 accessions introduced from International Center for Tropical Agriculture (CIAT). The breeding lines were developed by the Rwanda Agriculture and Animal Resources Development Board (RAB). This population consists of 96 bush and 96 climbers. The accessions of which id start from B are bush and those of C are climbers.

### Plant growth conditions

The field experiments were conducted in Rwanda during 2019 season A and B in Rubona site (S 02° 29.014ʹ E 029o 45.962ʹ 1688 mas with granite soil parental material) and 2020 season A and B in Muhanga (S 02° 4.12ʹ E 29o 43.24ʹ 1865 mas with schist soil parental material) and Ngoma (S 02° 09.548ʹ E 030o 30.928ʹ 1674 mas with mix schisto-quartizitic rock as soil parental material) sites. The crop rotation was maize in Rubona and Muhanga, whereas soybean was the crop rotation in Ngoma. The experiments were established in an incomplete block design with three replications. The plot size was 4 rows, each 2 m long, with an inter-row spacing of 0.50 m. Mineral fertilizer was applied at recommended rates of 50 kg ha^–1^ of DAP (diammonium phosphate) combined with 25 MT ha^–1^ of organic manure at planting and 50 kg ha^–1^ of urea at weeding as top dressing. Organic fertilizers used differed in all sites; in Rubona, manure prepared with cow dung was used; in Muhanga, compost made of harvesting residues was used; and in Ngoma, manure from chicken was used. Weeds were controlled using a hand hoe, reflecting farmers’ common practices in Rwanda. Plants used for DNA extractions were grown in the growth room under the normal fluorescent lights until they started to generate true leaves to harvest during the winter season of 2021–2022 in Niigata University. Plants used for RNA extractions were grown in an outdoor net house at Niigata University during the summer and autumn seasons from May to November in 2024.

### Phenotyping for grain Fe and Zn concentration, growth habit and seed color

The Fe and Zn concentration of seeds were determined using an X-ray Fluorescence (XRF) spectrometry established in the RAB-Rubona center, as described in Mukamuhirwa and Rurangwa (2018). For each accession, three measurements were performed using samples taken from plants grown in three different plots in the field. Ten well-packed, soil-free pods were randomly selected and harvested from those attached to the middle of the plant body, rather than the uppermost or lowest part of the plant body. From the pods, seeds were harvested for each accession, then cleaned with distilled water, and placed in fresh paper bags. The surface of the sampled seeds was further cleaned by rubbing them for 60 seconds between two clean cloths soaked with distilled water. After being oven-dried at 60°C for at least 12 hours, each sample was ground in a coffee grinder EM0480 (Sunbeam, Sydney, Australia). Between samples, the inside of the grinder was carefully cleaned using a brush and vacuum cleaner. For XRF analysis, grounded powder of 10 g for each sample was scanned for 100 seconds while the sample cup was spun to analyze the sample’s iron and zinc concentration, and the intensity of the X-rays that were emitted was recorded (Mukamuhirwa and Rurangwa 2018).

Growth habit and primary seed color were phenotyped according to the Trait Dictionaries for Fieldbook Development (https://cropontology.org/). A qualitative description of the growth habit of the plants was determined based on visual classification using 1–5 categories: 1 = determinate bush; 2 = indeterminate bush habit with erect stems and branches; 3 = indeterminate bush habit with weak mainstem and prostrate stem and branches; 4 = indeterminate climber habit with weak, long, and twisted stem and branches; and 5 = determinate climber, none of the studied accessions belonged to category 5, though. Primary seed color was visually evaluated for predominant seed color following the color scale (1–9), where 1 = white, 2 = cream-beige, 3 = yellow, 4 = brown-maroon, 5 = pink, 6 = red, 7 = purple, 8 = black, and 9 = others.

### Double digested Restriction Site Associated DNA Sequence (ddRAD-seq) and GWAS

DNA samples were extracted from the 192 accessions using Nucleospin Plant II (Macherey-Nagel, Düren, Germany). Using the DNA extracts, ddRAD-seq was performed following Shirasawa *et al.* (2016). For the library construction, two restriction enzymes, *Pst* I and *Msp* I (FastDigest restriction enzymes; Thermo Fisher Scientific, MA, USA) were used to double-digest the genome DNA. The libraries were sequenced using a DNBSEQ-G400RS (MGI Tech, Shenzhen, China) in paired-end 251 bp mode. Data processing was performed using the supercomputer system services of the Japanese National Institute of Genetics (NIG, https://sc.ddbj.nig.ac.jp/en/guides/overview). The adaptor sequence was removed using cutadapt (Martin 2011) and the trimmed fastq files were mapped to the reference genome *Phaseolus vulgaris* v2.1 (Pvu2.1, https://phytozome-next.jgi.doe.gov/info/Pvulgaris_v2_1) using bowtie2 (Langmead and Salzberg 2012, Langmead *et al.* 2019) with default setting. Samtools (Danecek *et al.* 2021) was used for conversion from sam to bam files and SNP calling to generate the bcf files. Using bcftools (Danecek *et al.* 2021), the bcf files were converted to vcf files, then SNPs were filtered with VCFtools (Danecek *et al.* 2011) with the following options; --min-alleles 2 --max-alleles 2 --remove-indels --minQ 999 --minDP 5 --max-missing 0.8 --maf 0.03. Among the SNP sites, only those with a heterozygosity of 20% or less among the 192 accessions were selected using Tassel V5 (Bradbury *et al.* 2007). Finally, 8,480 SNPs were screened for the downstream analyses. The read data was deposited to DDBJ BioProject with accession number PRJDB19846.

The phylogeny analysis of the population and GWAS were conducted as follows. Using the VCF file containing information of the 8,480 SNP sites, a neighbor-joining (NJ) tree was built and saved as a newick format file using “create tree” function of Tassel V5. Then the R package ggtree (Xu *et al.* 2022) was used to draw the unrooted tree. For GWAS, Mixed-Linear Model (MLM) method was used with Q + K model (Liu *et al.* 2016) using Tassel V5. For the value of K, the number of the subpopulations, the results obtained using ADMIXTURE (Alexander and Lange 2011) was used, while Q, the population structure was calculated using Tassel V5. The results of the association analysis were corrected for multiple testing using the Bonferroni adjustment, considering the alpha value of 0.05. Haploview (Barrett *et al.* 2005) was used to draw Linkage disequilibrium (LD) structure at the genome region highly associated with seed Zn concentration.

### Acquisition of the candidate genomic region sequence for seed Zn in C90 accession

We sequenced the genome of C90, one of the 192 accessions of high seed Zn phenotype (see details in Results), following the protocol described by Naito (2023). Briefly, we extracted DNA of C90 from the leaves using Nucleobond HMW DNA Kit (Macherey-Nagel), then used it for library preparation with Ligation Sequencing Kit SQK-LSK114 (Oxford Nanopore Technologies, Oxford, UK). The library was sequenced with an PromethION Flow Cell R10.4.1 (Oxford Nanopore Technologies) in PromethION 24 (Oxford Nanopore Technologies). The obtained long-read sequences were assembled using NECAT (Chen *et al.* 2021) polished once with medaka-1.9.1 (https://github.com/nanoporetech/medaka) with -g option. BUSCO (Benchmarking Universal Single-Copy Orthologs) score of the genome assembly was calculated by BUSCO v5.8.0 using fabales_odb10 as datasets (Manni *et al.* 2021). The sequence in C90 corresponding to the candidate region for seed Zn concentration defined on Pvu2.1 was identified using blastn (Camacho *et al.* 2009) as follows. A blastn search using two 300 bp-long sequences at the both ends of the candidate region defined on Pvu2.1 (Chr11: 5,288,249–5,288,548 and Chr11: 5,698,199–5,698,498) as queries and C90 genome assembly as database identified the top-hit subject sequences separated by approximately 400 kb interval on the same contig. This region was retrieved as the sequence of the candidate region in C90. AUGUSTUS (Sommerfeld *et al.* 2009) was used to predict genes in this candidate region. The long-read data was deposited to DDBJ BioProject with accession number PRJDB19846.

### PCR genotyping and sequencing

We conducted PCR genotyping of the three candidate genes for seed Zn concentration using genome DNA used for ddRAD-seq. For *Phvul.011G060000* and *Phvul.011G061200*, PCR products were electrophoresed on the agarose gels to separate amplicons of different molecular sizes. For *Phvul.011G058500*, PCR products were digested with *Pvu* I (New England Biolabs, MA, USA), then electrophoresed on agarose gels to detect presence-absence of the recognition site in the amplified sequence introduced by the dCAPs primer. We used EmeraldAmp MAX PCR Master Mix (TaKaRa Bio, Shiga, Japan) for these PCR. The amplified products from selected accessions were gel purified using FastGene Gel/PCR Extraction Kit (Nippon Genetics, Tokyo, Japan), then sequenced by Fasmac Co., Ltd., Kanagawa, Japan. The primers used these experiments are listed in Supplemental Table 2.

### RNA extraction and quantitative RT-PCR (qRT-PCR)

Two accessions, B76 and C54, were selected from the 192 accessions for qRT-PCR experiments as representative accessions of low and high seed Zn concentrations, respectively, because these accessions had typical haplotypes for the locus highly related to seed Zn concentration (see details in Results). We harvested leaves and roots of one-month old plant, and pod wall (pericarp), seed coats and embryos at the stage when immature seeds reached at the maximum size. These samples were collected from three plants of each accession. From these samples, total RNA samples were extracted following the method described in Fukai *et al.* (2010). Then we synthesized cDNA from the RNA with PrimeScript RT-PCR Kit (TaKaRa Bio) using mixture of oligo dT primer and random hexamers following the manual. qRT-PCR was conducted using LightCycler Nano (Roche, Basel, Switzerland) and FastStart Essential DNA Green Master Mix (Roche). *Actin11* (*Phvul.008G011000*) was used as the internal control. The primers used in this analysis are listed in Supplemental Table 2.

## Results

### Phenotypic diversity in 192 accessions of RAB germplasms

**Fig. 1.**
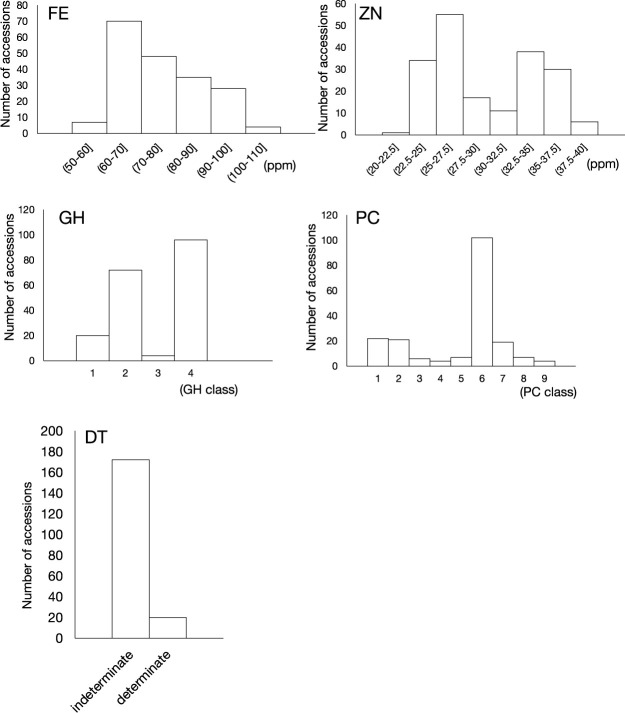
Frequency distribution of 192 common bean accessions for five traits. FE: iron concentration in dried seeds, ZN: zinc concentration in dried seeds, GH: class of growth habitat, PC: class of primary seed color, DT: determinate or indeterminate.

The 192 accessions used in this study was those stored and/or bred in RAB. The half of the population was composed of climbers (vine-like requiring supports such like stalks), and the other half was composed of bush (standing on their own). According to the CIAT definition of phenotypic classification, the growth habits of the accessions were classified into 4 classes. All climbers belong to class 4, while bush belong to either of class 1, 2 and 3 (see details in Materials and Methods). Among them, only class 1 has determinate growth habit. The following five traits were investigated in this study, that is, seed Fe concentration (FE), seed Zn concentration (ZN), growth habit (GH), shoot determinacy (DT) and primary seed color (PC). Among them, FE and ZN are quantitative traits but others are qualitative traits. These traits were observed six times, two seasons at three sites in Rwanda (see details in Materials and Methods). Supplemental Table 3 shows the trait values recorded at each observation, and averages between observations. Since three qualitative traits were always constant and not affected by both sites and seasons, there is only one data column for each of them. Supplemental Table 4 shows the correlations between traits of all possible combinations. The mean correlation among values of ZN in the six datasets was 0.68, with 14 of the 15 comparisons exceeding 0.6, suggesting the high degree of stability of this trait to environmental conditions. On the other hand, correlations between FE varied from 0.17 to 0.75, and the average was 0.5 (Supplemental Table 4), still high but lower than ZN. We noticed that correlations of FE between three datasets of season A were higher than those of season B, suggesting season dependency and better heritability in season A than B for FE. Analysis of variation (ANOVA) revealed the high level of proportion of variance explained (PVE) by accession was 57.16% for ZN and 46.43% for FE, with ZN being higher than FE (Supplemental Tables 5, 6). On the other hand, PVEs due to the combination of accession and season (“Accession:Season” in Supplemental Tables 5, 6) and accession and site (Accession:Site in Supplemental Tables 5, 6) were both higher for FE than ZN (10.08% vs 3.19% and 17.01% vs 10.62%, respectively). Above all, the data revealed that FE tend to be more easily influenced by growth conditions compared to ZN. Therefore, we decided to use the average value from three datasets of season A as FE and average value from all six datasets as ZN for following GWAS. The correlation between FE (average of season A) and ZN (average of all datasets) was 0.48 (Supplemental Table 4). Both FE and ZN deviated from a normal distribution (Fig. 1) with ZN showing a bimodal distribution.

### Phylogenic relationship and population structure among the Rwandan common bean accessions

**Fig. 2.**
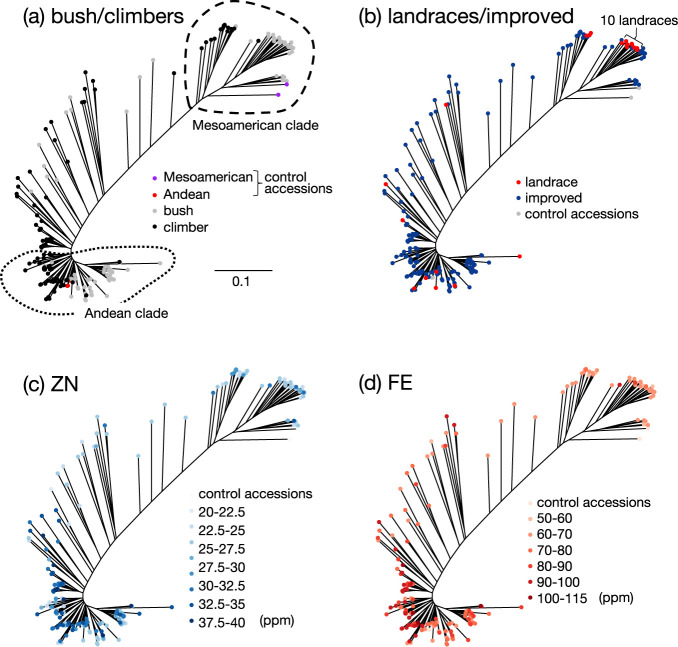
Relation between phylogeny and traits among the 192 accessions. The unrooted phylogenic tree was drawn based on the 6,383 SNPs among the 197 accessions and five accessions, two Mesoamerican and three Andean accessions previously investigated (Cortinovis *et al.* 2024). The ends of the branches were colored according to (a) bush and climber classification, (b) landraces or improved accessions, (c) zinc concentration in dried seeds, (d) iron concentration in the dried seeds. In (a), Mesoamerican and Andean clades were indicated. In (b), a group constituted of 10 landraces was indicated.

ddRAD-seq and the following filterization steps resulted in the identification of 8,480 SNPs among the 192 accessions that were used for further analyses. Based on the SNPs, the population structure was investigated using ADMIXTURE (Alexander and Lange 2011). The CV value decreased with increasing K, the number of subpopulations, and once reached minimum at six, however, continued to decrease when K was equal to or above eight (Supplemental Fig. 1a). PCA of genetic diversity in the population showed slow increase of cumulative proportion in the genetic variance following the increase in the number of components (Supplemental Fig. 1b), and when number of components was six, 60.1% of the genetic variance can be explained (Supplemental Fig. 1c). From these results, we decided to use six as the number of subpopulations in the GWAS described later. The diagram of the population structure clustered by K = 6 showed complex genetic relationship among the accessions (Supplemental Fig. 1d). An unrooted phylogenetic tree was drawn to know the genetic relationship among 192 accessions. The tree was drawn based on the information of 6,386 SNPs identified among the 197 accessions, including two Mesoamerican and three Andean accessions that were previously investigated by Cortinovis *et al.* (2024), addition to the 192 Rwandan accessions. The tree had two large clades at both ends, each corresponding to Mesoamerican and Andean, connected by the intermediate accessions (Fig. 2a). In the phylogenic tree, several grouops mainly composed of either bush or climbers were found, suggesting the constraints on genetic exchange between the two subpopulations in the Rwandan population. The phylogenic tree colored with GH clearly showed that among the three groups in the Mesoamerican clades, one of them was almost exclusively composed of climbers and the other two were composed of bush (Fig. 2a, Supplemental Fig. 2a), again supported the idea of growth-habit based genetic barrier. The accessions having determinate meristem, corresponding to class 1 of GH, tend to be found in the clade occupied by class 2 (Supplemental Fig. 2a, 2b), possibly suggested the evolution of class 1 from class 2 by small number of mutations controlling this trait. We noticed that 10 landraces generated a group in Mesoamerican (Fig. 2b), and nine out of the 10 landraces are bush (Fig. 2a, 2b). This group might represent a traditional type of bush in Rwanda. Interestingly, both FE and ZN showed association with phylogeny, that is, accessions with high FE and ZN values were more often found in Andean than in Mesoamerican in this population (Fig. 2c, 2d), reflecting the relatively high level of correlation between them (Supplemental Table 4). On the other hand, no obvious association was found between PC and phylogeny (Supplemental Fig. 2c).

### GWAS

**Fig. 3.**
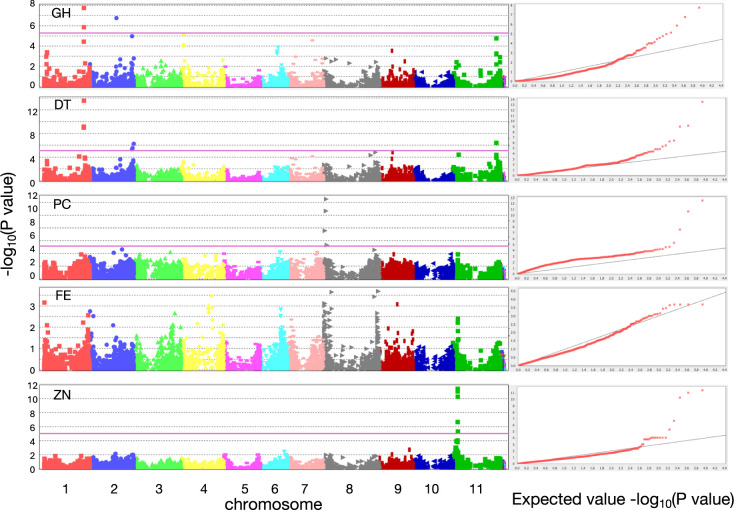
Manhattan plots and qq plots of five traits. FE: iron concentration in dried seeds, ZN: zinc concentration in dried seeds, GH: class of growth habitat, PC: class of primary seed color, DT: determinate or indeterminate. In manhattan plots, horizontal purple lines indicate threshold when Bonferroni correction at α = 0.05 was set up.

GWAS was performed to identify loci controlling the investigated agronomic traits. Regarding the two qualitative traits related to plant structure, GH and DT, the common significant SNPs were identified at the 44,808,951 and 44,715,304 on chromosome 1 (Fig. 3, Table 1). Addition to that, one GH-specific and two DT-specific significant SNPs were detected (Fig. 3, Table 1). *PvTFL1y* (*Phvul.001G189200*), a regulatory gene for shoot meristem in common bean (Kwak *et al.* 2008, Repinski *et al.* 2012), was found 47 kb upstream from the common significant site at 44,808,951 on chromosome 1, suggesting that the gene could be responsible for this locus, and the large influence of this gene to the growth habit in the Rwandan population. For PC, two SNPs close each other, 3,023,090 and 3,225,956 on chromosome 8, were detected as significant (Fig. 3, Table 1). There is a cluster of *MYB* genes in the vicinity of these SNPs, at least one of which, *Phvul.008G038400*, is known to be involved in the regulation of seed coat color, particularly anthocyanin biosynthesis (Table 1, Campa *et al.* 2023, García-Fernández *et al.* 2021). The genome-wide average of half decay of linkage disequilibrium (LD) between SNPs used in this study was relatively large at 2.2 Mb (Supplemental Fig. 3). Nevertheless, the detection of significant SNPs within a few 10 Kb of known genes for GH/DT and PC indicates that this GWAS was performed properly and that where significant SNPs were detected for a trait, the physical distance between the SNP and the causal polymorphism was relatively close. However, no significant peaks for FE were detected (Fig. 3), even though we used average values of Fe concentration among three season A datasets for FE because of the less environmental influence. In addition, we conducted GWAS using six seed Fe concentration datasets independently, again no significant peaks were detected. For ZN, on the other hand, a series of neighboring five SNPs on chromosome 11 were detected as significant (Fig. 3, Table 1). Possible reasons why no significant SNPs were detected for FE could be that the trait is regulated by many loci, or that the SNP density at the major responsible loci was low.

**Fig. 4.**
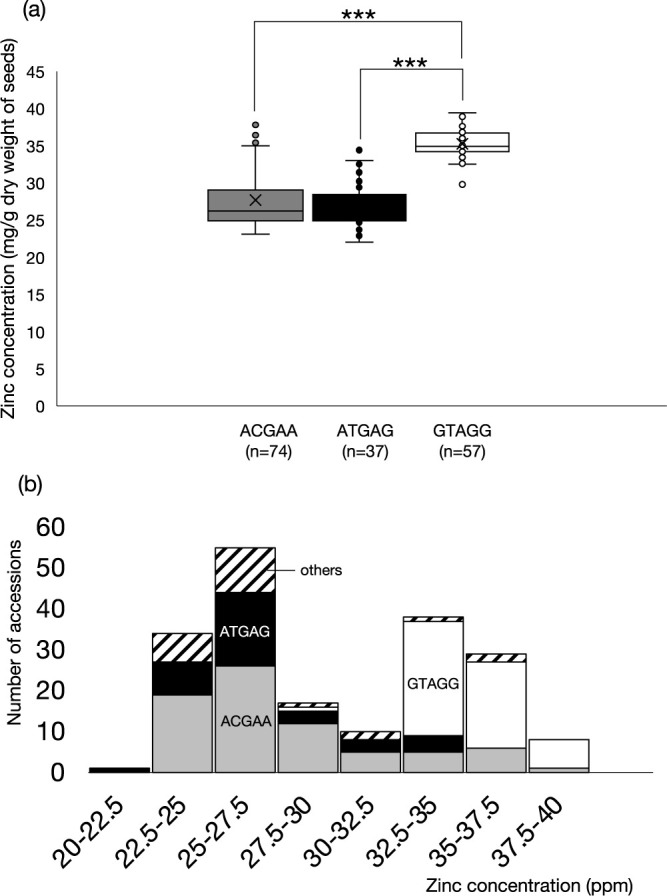
Association between seed zinc concentration and haplotype at ZN locus on chromosome 11. (a) Comparison of zinc concentration between three main haplotypes. Paire t-test between GTAGG and other two haplotypes suggeseted significant difference between them. ***, p < 0.0001. (b) Haplotype distribution and frequency distribution of seed zinc concentration among the 192 accessions.

We could classify the 192 accessions according to the haplotypes defined by the five SNPs. There were 14 haplotypes (including haplotypes possessing hetero calls) among the 192 accessions, however, 168 of them (87.5%) belonged to one of the three haplotypes, ACGAA, ATGAG and GTAGG (Supplemental Table 7). We found correlation between ZN and the three haplotypes, that is, GTAGG haplotype had significantly higher average ZN (35.3 ppm) compared to the other two (ACGAG; 27.7 ppm, ATGAG; 26.9 ppm, Fig. 4). The haplotype at this locus in the reference genome *Phaseolus vulgaris* v2.1 (Pvu2.1, DOE-JGI and USDA-NIFA, https://phytozome.jgi.doe.gov/) was ACGAA, one of the three major haplotypes found in this study associated with low ZN. Haploview (Barrett *et al.* 2005) detected three linkage blocks in this genomic region, with four of the five significant SNPs being in a 278 kb block and another in a 336 kb block (Supplemental Fig. 4).

### The three candidate genes for ZN

The significant five SNPs for ZN localised within 210-kb region from 5,388,249 to 5,598,498 on chromosome 11. The SNP with highest –log_10_(P-value) was detected at 5,397,233. In the cases of GH/DT and PC described above, the known genes regulating the traits were found within 50-kb region of the closest significant SNPs. Therefore, we defined the candidate region for ZN from 5,288,249 to 5,698,498 on chromosome 11, from 100 kb (twice as large as the distance between *PvuTFL1y* and the closest significant SNP) upstream of the first SNP to 100 kb downstream of the last SNP, spanning 410,250 bp. In this region, 47 genes have been annotated in Pvu2.1. Since the haplotype at this locus in Pvu2.1 is ACGAA, one of the two haplotypes representing low ZN phenotype (Fig. 4), the genome sequence information could be a representative of a low ZN accession. To know the genome information of the high ZN accession, we conducted longread sequencing of C90, an accession with high ZN (33.6 ppm) having GTAGG haplotype. Using Nanopore sequencing, we obtained 559 Mb-long genome assembly with BUSCO score of 99.2%. (Supplemental Table 8). From the genome assembly, the 420,707 bp-long region was identified corresponding to the candidate region using blastn (see details in Materials and Methods). Vista plot (Frazer *et al.* 2004) showed high level of conservation of the genome structures between the two alleles (Supplemental Fig. 5). From this region of C90, AUGUSTUS predicted 56 genes excluding transposons, of which 47 genes were annotated in the allelic region of Pvu2.1. As for the remaining nine C90-specific genes, we assumed them to be incorrectly predicted genes, as none of them possess functional domains or conserved structures. Next, we compared the sequences of the 47 genes between the two alleles. The search revealed that CDSs of 18 genes were totally identical, 6 genes had only synonymous SNPs, and the other 23 had polymorphisms altering protein sequences between the two alleles (Supplemental Table 9). Among them, four genes, *Phvul.011G059201* (*Phvul.011G059201.1*), *Phvul.011G060300* (*Phvul.011G060300.1*), *Phvul.011G060600* (*Phvul.011G060600.1*) and *Phvul.011G063100* (*Phvul.011G063100.2*) had large structural differences in their encoded protein sequences between the alleles. Among the four genes, *Phvul.011G063100*, encoding a GRAS family transcription factor, the Pvu2.1 allele seems to be a mutant allele, since the encoded protein sequence is shorter than that of C90 allele that has authentic structure of GRAS transcription factor (Supplemental Table 9). On the other hand, C90 allele of *Phvul.011G060300*, encoding a UDP-glucosyl transferase, seems to be a mutant allele, since the encoded protein is shorter than that of Pvu2.1. The other two genes among the four did not have functional annotations (Supplemental Table 9). Altogether, it was difficult to screen the candidate genes only from their allelic polymorphisms. Next we tried to identify candidates based on the functional annotations of the 47 genes, and found two genes, *Phvul.011G060000* encoding a Multidrug and toxin extruder (MATE) efflux family protein 1 and *Phvul.011G061200* encoding a Metal tolerance protein B (MTP) (Supplemental Table 9). In addition, *Phvul.011G058500* encoding a ZIP transporter, was found 56-kb upstream of the candidate genome region (Supplemental Fig. 4). Izquierdo *et al.* (2018) detected a QTL regulating both ZN and FE on the genome region which is close to the ZN region of this study by meta-QTL analysis, and suggested *Phvul.011G058500* as a candidate. Therefore, we added this gene as a candidate even though it is outside of our focused genome region (Supplemental Fig. 4).

In *Phvul.011G058500*, there were no polymorphisms in both exons and introns between Pvu2.1 and C90 alleles. At 393-bp upstream of the translation start site of this gene, C90 had an insertion of 8 bases compared to Pvu2.1. However, analysis using a dCAPs primer detecting this indel revealed that none of the 192 accessions had Pvu2.1 allele. Both sequences of the PCR products of this region from C85, having ACGAA haplotype same as Pvu2.1, and C90, having GTAGG haplotype, were totally identical each other (Supplemental Fig. 6), agreed with the results obtained by the dCAPs analysis. From the dCAPs experiments, PCR product approximately 50 bp longer than expectation (308 bp and 316 bp from Pvu2.1 and C90, respectively) was amplified from 34 accessions. We designated the allele from which short PCR fragment was amplified as *Zip_l*, and the allele from which long fragment was amplified as *Zip_h* (Supplemental Table 7). Sequencing the PCR products revealed that the product from *Zip_h* had a 41 bp insertion at the site of the indel, resulting the amplified length as 349 bp (Supplemental Fig. 6).

*Phvul.011G060000* (*MATE efflux family protein*) had eight SNPs on CDS between the Pvu2.1 and C90 alleles. Three of which were non-synonymous substitutions (Supplemental Fig. 7, Supplemental Table 9). One of them was in the cytoplasmic domain on the N-terminal of the MATE efflux protein, and the other two were in the region where the transmembrane domains and the cytoplasmic domains appeared (Supplemental Fig. 7). The introns are highly polymorphic between the two alleles. In particular, the lengths of the eighth intron significantly differed, 616 bp in Pvu2.1 and 562 bp in C90, enabled us to design the primers distinguishing the two alleles (Supplemental Figs. 8, 9). However, the genotyping identified three alleles based on the amplified fragment length. The first one was designated as *MATE_l* corresponding to C90 allele of which length was 302 bp, the second one was designated as *MATE_h* corresponding to Pvu2.1 allele of which length was 356 bp, and the third one was designated as *MATE_m* of which length was intermediate between *MATE_l* and *MATE_h*, 309 bp (Supplemental Fig. 9).

Regarding the third candidate gene *Phvul.011G061200* (*Metal tolerance protein B1*), two splicing variants were annotated as *Phvul.011G061200.1* and *Phvul.011G061200.2* in Pvu2.1. *Phvul.011G061200.1* encodes a protein of 389 amino acid residues while *Phvul.011G061200.2* encodes a shorter protein of 291 residues. This is because Phvul.011G061200.2 lacks the C-terminus region present in Phvul.011G061200.1 (Supplemental Fig. 7). According to the annotation by InterPro (Paysan-Lafosse *et al.* 2023), the region presents in Phvul.011G061200.1 but absent in Phvul.011G061200.2 has a feature of Cation efflux CTD superfamily (Supplemental Fig. 7). Four SNPs on the CDS of *Phvul.011G061200.1* were found between Pvu2.1 and C90, one of which was a non-synonymous substitution (Supplemental Fig. 7, Supplemental Table 9). This non-synonymous substitution is located at residue 43 from the N-terminus, within the non-cytoplasmic domain and is common to both splicing variants. The 5ʹ upstream of the genes was highly polymorphic between Pvu2.1 and C90, enabled us to design the primers distinguish the two alleles (Supplemental Figs. 10, 11). Based on the amplified length, we could distinguish between C90 and Pvu2.1 alleles and designated them as *MTP_l* and *MTP_h*, respectively (Supplemental Table 7). The strong associations between genotypes of the two genes, *Phvul.011G060000* and *Phvul.011G061200*, and the three major haplotypes defined by the five significant SNPs were observed. That is, ACGAA haplotype always linked with *MATE_h* and *MTP_h*, while GTAGG haplotype always linked with *MATE_l* and *MTP_l*, and ATGAG haplotype always linked with *MATE_m* and *MTP_h* (Supplemental Table 7). On the other hand, genotype of *Phvul.011G058500* showed imperfect association with the haplotypes (Supplemental Table 7), suggesting the weaker linkage between this gene and the ZN region detected by GWAS, in spite of the relative short physical distance from the closest significant ZN SNP (Supplemental Fig. 4). Therefore, we decided to conduct qRT-PCR to see the levels of transcripts of *Phvul.011G060000* and *Phvul.011G061200* in the following section.

### Expression analysis of candidate genes by qRT-PCR

**Fig. 5.**
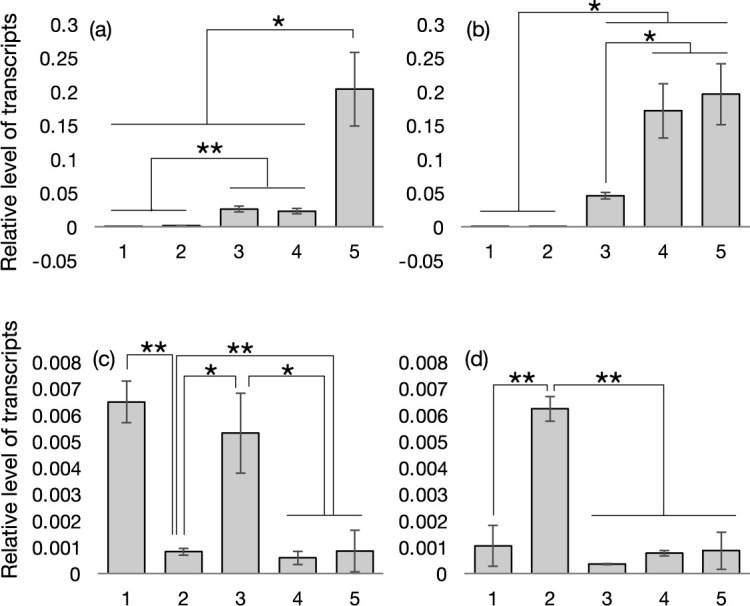
Relative levels of transcripts of *Phvul.011G060000* (a, b) and *Phvul.011G061200* (c, d) revealed by qRT-PCR in B76 (a, c) and C76 (b, d) accessions. The numbers indicate tissues, 1; leaves, 2; roots, 3; embryos, 4; seed coats, 5; pod wall (pericarp). Data are expressed as means ± standard deviations derived from triplicates. Significant differences were detected with p < 0.05 and **, p < 0.01 by Student’s T-test.

We conducted qRT-PCR using two accessions, B76 and C54. B76 has low ZN phenotype and ACGAA haplotype with *MATE_h* and *MTP_h*. C54 has high ZN phenotype and GTAGG haplotype with *MATE_l* and *MTP_l*, as C90. In both accessions, *Phvul.011G060000* (*MATE efflux family protein*) showed higher level of transcripts in the three pod-related tissues compared with two vegetative tissues (Fig. 5a, 5b), suggesting. the function of this gene in floral tissues during seed development. On the other hand, *Phvul.011G061200* (*Metal tolerance protein B1*) showed different patterns of transcript accumulation between the two accessions. The level was highest in the roots compared to other tissues in C54, while the level was low in roots, but higher in leaves and embryos in B76 (Fig. 5c, 5d).

## Discussion

In this study, we conducted GWAS of five agronomic traits using 192 accessions of common bean possessed and maintained by RAB. We could detect SNPs significantly associated with four traits, however, no sites for FE were identified. The genome region highly associated with ZN was identified on chromosome 11, and three candidate genes in the region were analyzed in detail. Among the three genes, we focused on the two genes because of the strong association with haplotypes defined by the five significant SNPs of ZN detected by GWAS. One of them, *Phvul.011G060000* showed similar tissue specificities, while the other gene, *Phvul.011G061200* showed different tissue specificities between the two accessions investigated. It should be noted that the Rwandan common beans growing in Niigata, Japan, experienced significant flowering delays and high temperature-related stresses, and low fertility. These may have affected the material used for qRT-PCR. Therefore, it is necessary to verify whether the results of the qRT-PCR are reproducible in plants grown under the optimal growing conditions.

In Arabidopsis, AT1G51340 showed the highest homology to Phvul.011G060000, and the second highest homolog was AT3G08040, known as AtFRD3 (*A. thaliana* ferric reductase defective 3). It is known that AtFRD3 is necessary for citrate transport to xylem that is necessary for Fe transfer from the roots to the shoots (Durrett *et al.* 2007). AtFRD3 is also expressed in floral organs and necessary for seed development since the loss of function mutants showed seed sterility (Roschzttardtz *et al.* 2011). Interestingly, Pineau *et al.* (2012) identified *AtFRD3* as a responsible gene for natural variation in Zn tolerance between accessions of Arabidopsis, suggesting the function as a regulator for cross-homeostasis between Fe and Zn. They also found that the allelic polymorphisms caused differences both at the transcript level and in citrate efflux activity when expressed in exogenous systems. Scheepers *et al.* (2020) showed that *atfrd3* mutant roots overaccumulated iron and zinc, exhibiting a transcriptome profile characteristic of iron-deficient plants, and that the abnormal phenotype of the mutant was alleviated in the presence of excess zinc. This suggests that metal homeostasis mediated by this MATE efflux protein consists of a complex network involving multiple signalling pathways. Since Phvul.011G060000, AT1G51340 and AtFRD3 are phylogenetically close to each other (Islam *et al.* 2022, Takanashi *et al.* 2014), their protein function is likely to be similar, the biological function of AT1G51340 has not been clarified yet, though. AT2G29410 showed highest homology to Phvul.011G061200, known as MTP4, and the second highest homolog was AT2G46800, known as MTP1 in Arabidopsis. MTP1 is a Zn transporter localised to vacuolar membrane, necessary for accumulation of excess Zn in vacuole and Zn torelance in plants (Desbrosses-Fonrouge *et al.* 2005). On the other hand, Muro *et al.* (2024) showed that MTP4 is highly expressed in pollen grains and localised on *trans*-Golgi network. However, no obvious abnormalities were observed in the loss of function mutant of *MTP4*. These information from Arabidopsis homologs suggest that spatiotemporal patterns of expression and subcellular localization are important for the function of these genes, and that allelic polymorphisms are involved in the diversification of the function of these genes.

This study also revealed genetic relationship in the Rwandan common bean population. It was turned out that the Rwandan population is composed of Mesoamerican and Andean gene pools and their admixtures, as observed in other populations (Bellucci *et al.* 2023, Lobaton *et al.* 2018, Wu *et al.* 2020). Eastern Africa, including Rwanda, is distant from the place of origin of this plant species. Therefore, past genetic bottlenecks could have a significant impact on the genetic structure of the plant species nowadays. Given that the collaborative breeding programs of international framework have been ongoing in Rwanda since 80s, at least some portion of the genetic diversity found in this study could be due to the period. On the other hand, the subpopulation containing ten Rwandan landraces, composed of nine bush and one climber, was identified in the Mesoamerican clade. This group could represent a traditional type of bush been grown in Rwanda. The phylogenic tree also revealed the hidden constraints of the genetic exchange between accessions of different types of GH in the Rwandan population. Since GH/DT would be controlled by a small number of loci having large effects (Fig. 3), DNA marker assisted selection would be very useful to screen desirable GH/DT from the segregating population between accessions having different growth habits. This will facilitate common bean breeding in Rwanda by overcoming the GH-dependent mating constraints.

Even though a number of attempts have been made to identify the loci controlling common bean seed ZN using GWAS, so far none of them identified significant SNPs at the locus of chromosome 11 where we identified in this study. In addition, in the previous studies, multiple peaks with significance of similar levels were often detected, rather than an almost unique peak being detected as was the case in the present study. On the other hand, the region close to our ZN region was detected as a locus associated with both ZN and FE by QTL studies using segregating populations between Mesoamerican and Andean parents (Izquierdo *et al.* 2018). Our data also revealed the relationship between FE and ZN and the phylogeny in the Rwandan population, that is, Andean tend to have higher seed concentration of these two minerals than Mesoamerican (Fig. 2c, 2d). Therefore, the ZN locus on chromosome 11 could explain the difference in seed Zn concentration between the two gene-pools in the Rwandan population. However, our data suggested that this locus is not highly associated with FE, unlike the results obtained from the past QTL analyses. The reasons for this inconsistency are still unknown. One reason for this may be differences in the growing conditions. Alternatively, the loci identified by us in this study and by Izquierdo *et al.* (2018) could be neighboring but different loci. In particular, it is possible that the *ZIP* gene, which they considered a candidate gene, had a small sequence polymorphism in our population, limiting its contribution to the trait. Higher correlation between FE datasets within season A compared to other combinations of datasets (Supplemental Table 4) suggested the presence of seasonal factors influencing seed Fe accumulation. It would be interesting to know the factors since this could be useful to control growth conditions to maximize the seed Fe concentration. Compared to FE, ZN was more robust as shown as higher correlation between different datasets (Supplemental Tables 4–6). If the ZN locus identified in this study is identical with those detected in the past QTL analyses (Izquierdo *et al.* 2018), since the growth environments between these studies were totally different, again ZN is likely to be a relatively stable traits, when the phenotypic differences are mainly derived from the phylogenetic differences between Mesoamerican and Andean gene pools.

For the molecular identification of the responsible gene for ZN, further analyses should be needed. However, the primers designed in this study for genotyping *Phvul.011G060000* and *Phvul.011G061200* could be readily used for DNA marker-assisted breeding to increase seed Zn concentration not only in Rwanda but also in other countries, potentially expanding the effectiveness of biofortification with common bean.

## Author Contribution Statement

Data collection, FM, KS, KN, ER, VN, AN, JPM, MG, NO and KO; data analysis, FM, KS, KN, MO and EF; accession collection, ER, AN, JPM; experimental design, FM, KS and EF; project management, MG and EF.

## Supplementary Material

Supplemental Figures

Supplemental Table 1

Supplemental Table 2

Supplemental Table 3

Supplemental Table 4

Supplemental Table 5

Supplemental Table 6

Supplemental Table 7

Supplemental Table 8

Supplemental Table 9

## Figures and Tables

**Table 1.** Detected significant SNPs for each trait

Trait*	Chromosome	Position	p-value	MarkerR2	Candidate gene
GH	1	44715304	1.38E-06	0.1457	
		44808951	1.78E-08	0.19802	*PvuTFL1y* (*Phvul.001G189200*, 44856138-44857862)
	2	30025748	1.74E-07	0.14796	
DT	1	44612462	1.04E-09	0.24214	
		44715304	7.08E-10	0.24713	
		44808951	2.88E-14	0.38807	*PvuTFL1y* (*Phvul.001G189200*, 44856138-44857862)
	2	47624881	4.85E-06	0.11577	
		48193983	3.11E-06	0.14192	
		49576259	5.70E-07	0.14016	
	11	48974241	3.99E-07	0.20312	
PC	8	1798116	2.75E-08	0.20434	
		3023090	2.25E-11	0.27712	
		3225956	3.07E-13	0.32928	*MYB-like DNA binding protein* (*Phvul.008G035800*, 3139572..3143006) *Transcription factor MYB113-related* (*Phvul.008G038200*, 3171371..3172441, *Phvul.008G038400*, 3188682..3190957, *Phvul.008G038500*, 3200639..3203064)
		4031604	4.91E-06	0.14105	
ZN	11	5388249	9.25E-12	0.29156	*MATE EFFLUX FAMILY PROTEIN 1* (*Phvul.011G060000*, 5384809..5389051)
		5397187	2.15E-07	0.16648	
		5397233	3.97E-12	0.30271	
		5456950	5.33E-11	0.29426	*METAL TOLERANCE PROTEIN B* (*Phvul.011G061200*, 5460628..5463026)
		5598498	4.89E-06	0.1335	

* GH: growth habit, DT: determinant or indeterminant, PC: primary seed color, ZN: seed Zn concentration, FE: seed Fe concentration
