# Supplementary material for: Genetic control of seed iron and zinc concentration in Rwandan common bean population revealed by the Genome Wide Association Study (GWAS)
Source: Breed Sci. 2025 Jun 18;75(3):187–99. doi: 10.1270/jsbbs.24087 (PMC12457783; doi:10.1270/jsbbs.24087)
Supplement: Supplementary file 1 — Supplemental Figures [file 75_187_s1.pdf]

Mukamuhirwa et al./Breeding Science
doi: 10.1270/jsbbs.24087

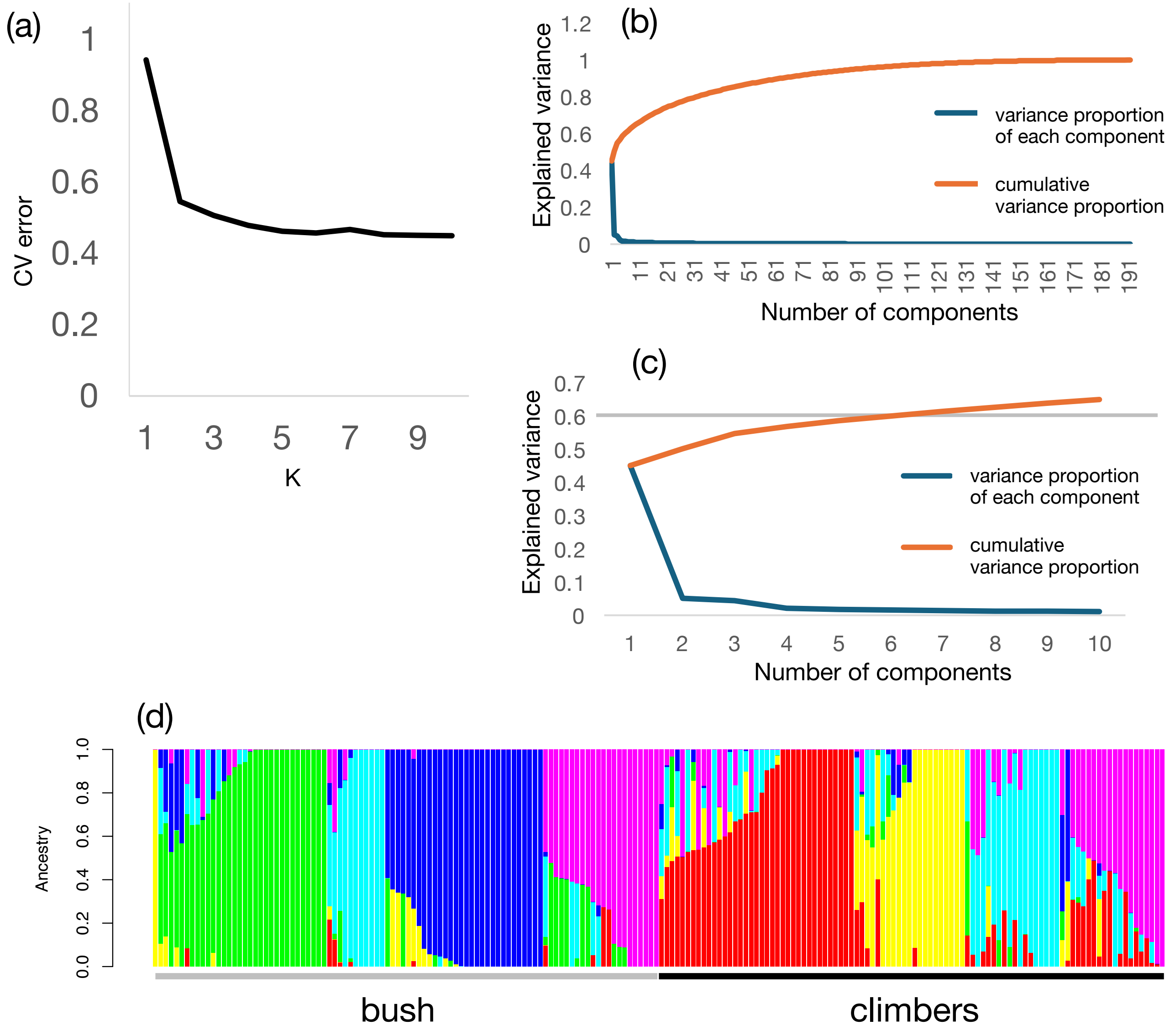

Supplemental Fig. 1 Population structure of the 192 common bean accessions.

(a) Cross-varidation error plot calculated by ADMIXTURE for K=1-10. (b) Genetic variance in the population explained by the number of components from 1 to 200. (c) Genetic variance in the population explained by the number of components from 1 to 10. 60% of total variance could be explained when number of components larger than six. (d) A coloured bar graph showing structure identity of the population estimated based on K=6. Left half indicates to bush and right half indicates climbers.

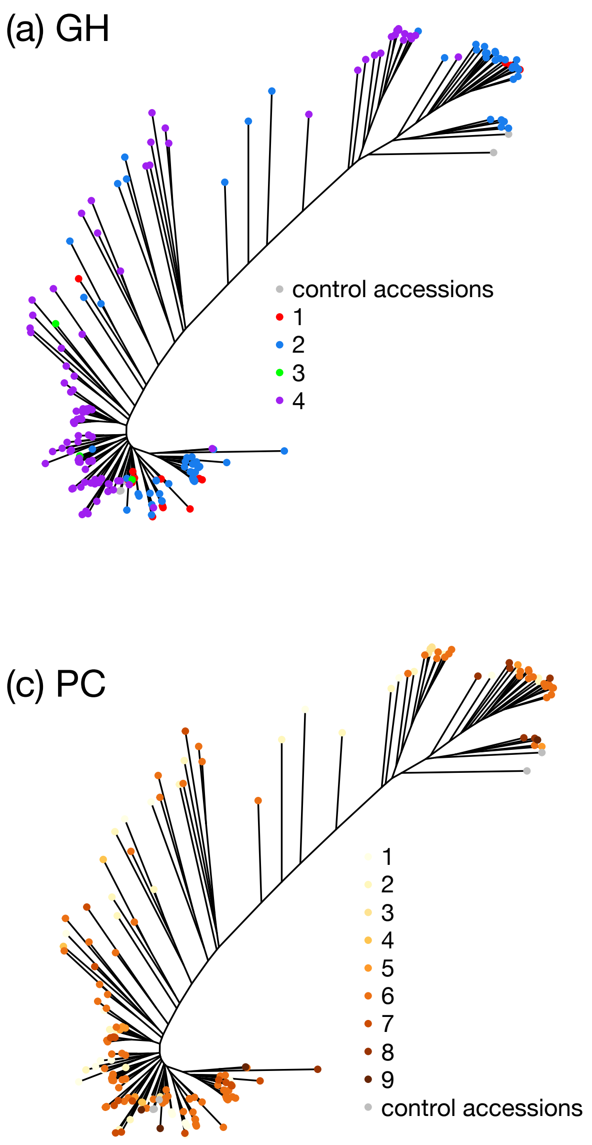

Supplemental Fig. 2 Relation between phylogeny and traits among the 192 accessions.

The unrooted phylogenic tree was drown based on the 6383 SNPs among the 197 accessions and five accessions, two Mesoamerican and three Andean accessions previously investigated (Cortinovis et al., 2024). The ends of the branches were coloured according to (a) class of growth habit, (b) Determinant or indeterminant, (c) class of primary colour of seed coat.

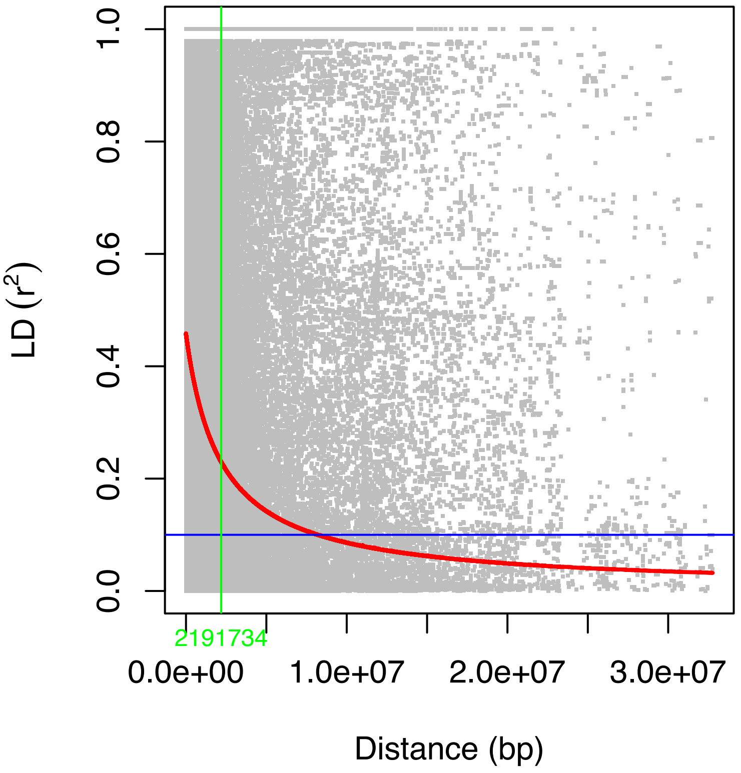

Supplemental Fig. 3 LD decay with physical distance.

Dots shows the r^2 among pairs of SNPs on the indicated physical distance. Red line shows the r^2 of non-linear least squares fit. Green line indicates that the half-decay LD distance is approximately 2.2Mb.

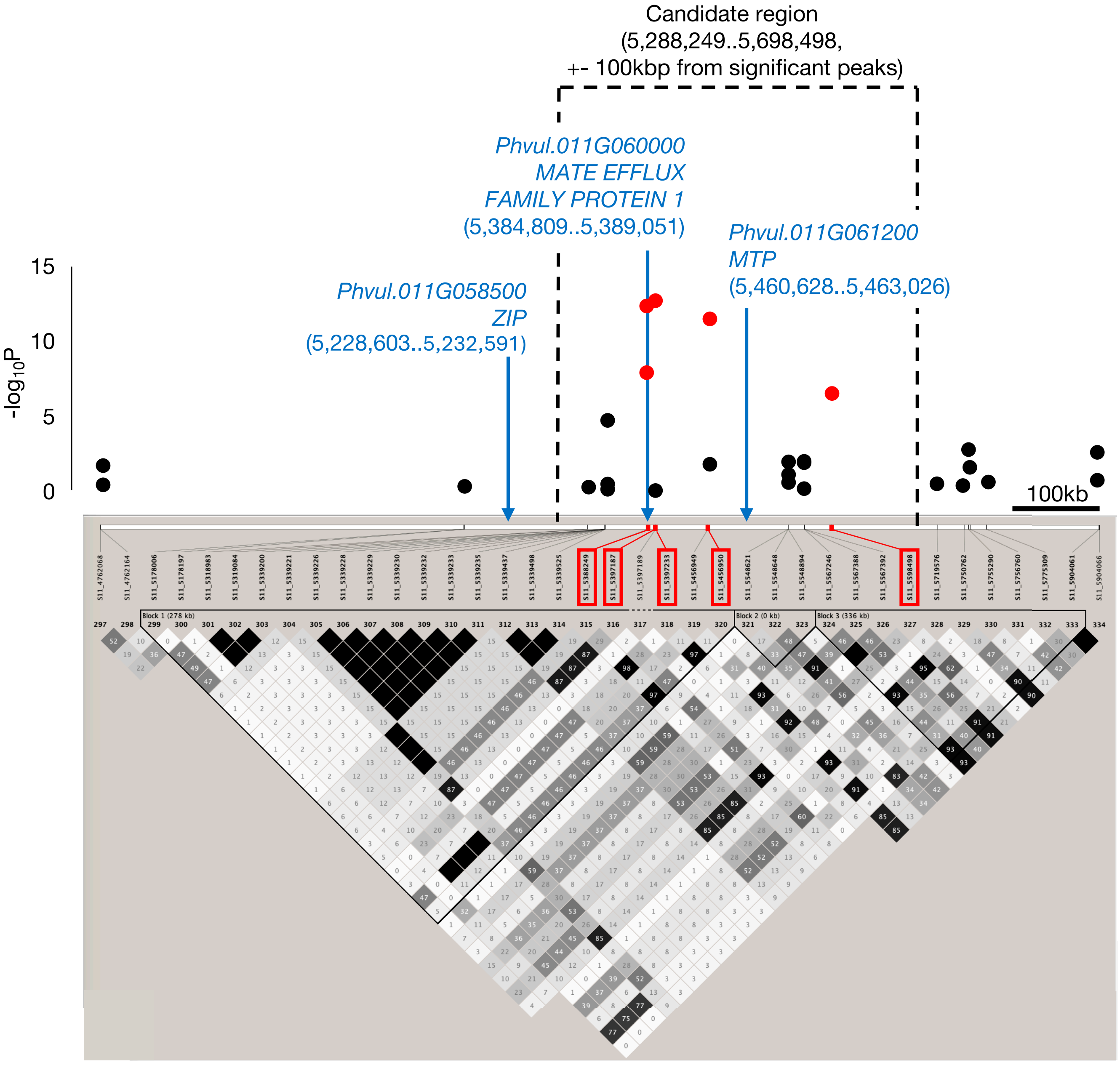

Supplemental Fig.4 A close-up view of Manhattan plot and LD structure at ZN locus.

The 38 SNPs identified in this region and their physical positions and $-\log_{10}P$ values are indicated above the heat map showing LD measured by r^2 (black; high r^2, white; low r^2). The five significant SNPs are marked with red. The candidate region from 5,288,249 to 5,698,498 is indicated with dotted line. The physical positions of the three candidate genes are indicated with blue arrows. The three linkage blocks detected by Haploview (Barrett et al. 2005) were shown as block 1, 2 and 3, respectively.

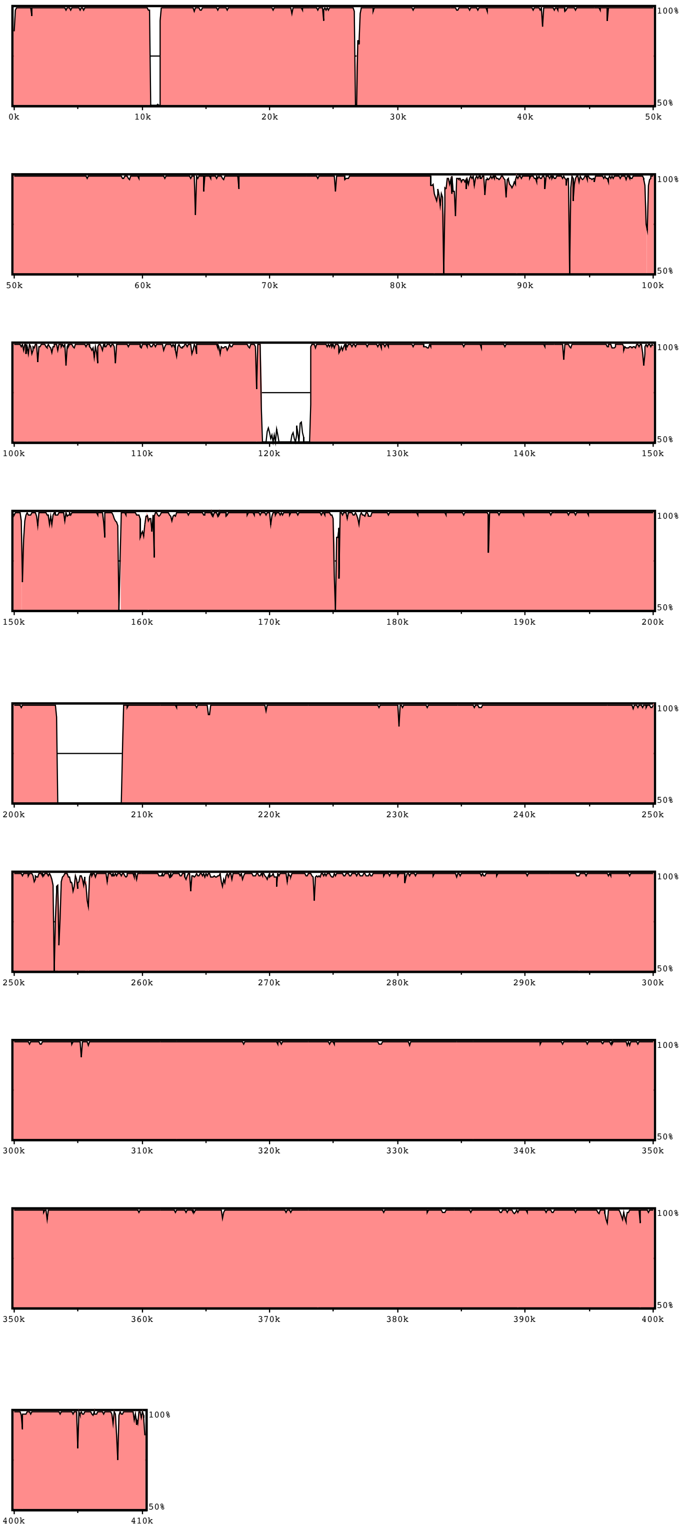

Supplemental Fig. 5 Vista plot between ZN locus between Pvu2.1 and C90 alleles.

The plot was drown using the genome sequence from 5,288,250 to 5,698,498 on chromosome 11 of Pvu2.1 as reference. Positions on the reference genome are indicated below the plot. The pink-coloured regions indicate structurally conserved region between the alleles while gapped regions indicate high level of polymorphism between the two sequences.

```
                         ZIP_dcapsF
Pvu2.1      AGCTTCTAAGCTCAATAAGATAGTCTTATAAATAAATGTTTAACCCAAGAGGTAAGATAA
C90/C85     AGCTTCTAAGCTCAATAAGATAGTCTTATAAATAAATGTTTAACCCAAGAGGTAAGATAA
B11         AGCTTCTAAGCTCAATAAGATAGTCTTATAAATAAATGTTTAACCCAAGAGGTAAGATAA
            ************************************************************

Pvu2.1      GTGAATTTATCTGATAATTATACAAAACTGATTTTAACTTTGTCATTGGAGTGCCATGCA
C90/C85     GTGAATTTATCTGATAATTATACAAAACTGATTTTAACTTTGTCATTGGAGTGCCATGCA
B11         GTGAATTTATCTGATAATTATACAAAATTGATTTTAACTTTGTCATTGGAGTGCCATGCA
            *************************** ********************************

Pvu2.1      GGTACATACTCACCCATGAGGAGAGAGCAGAATGAAAAAGAGACCAAAATGAAAAGGCAA
C90/C85     GGTACATACTCACCCATGAGGAGAGAGCAGAATGAAAAAGAGACCAAAATGAAAAGGCAA
B11         GGTACATACTCACCCATGAGGAGAGAGCGGAATGAAAAAGAGACCAAAATGAAAAGGCAA
            ****************************.*******************************

Pvu2.1      AAGATCTAATTGATCAAGCCCAGTTAAATAAGTTTGTCAGTCATCTAAGTCTCATATAAA
C90/C85     AAGATCTAATTGATCAAGCCCAGTTAAATAAGTTTGTCAGTCATCTAAGTCTCATATAAA
B11         AAGATCTAACTGATCAAGCCCAGTTAAATAAATTTGTCAGTCATCTAAGTCTCATATAAA
            ********* *********************.****************************

Pvu2.1      GTCAAAGAAAAAGTGAACCG----------------------------------------
C90/C85     GTCAAAGAAAAAGTGAACCGAAAGTATA--------------------------------
B11         GTCAAAGAAAAAGTGAACCGAAAGTATAATTCTCATATAAAGTGAAAGAAAAAGTGAATC
            ********************

Pvu2.1      ---------ATTGGAGAGAAAAACCGAAATCCCAATAATAGAAAGGAGTTTTGTTTATTA
C90/C85     ---------ATTGGAGAGAAAAACCGAAATCCCAATAATAGAAAGGAGTTTTGTTTATTA
B11         GAAAGTATAATTGGAGAGAAAAACCGAAATCCCAATAATAGAAAGGAGTTTTGTTTATTA
                     ***************************************************
                                                   ZIP_PvuI_region_R
Pvu2.1      TTATGGAACTTTTGTGAATAAGTAACATACACAATACCCAAGCTAGGTGATATGTGG
C90/C85     TTATGGAACTTTTGTGAATAAGTAACATACACAATACCCAAGCTAGGTGATATGTGG
B11         TTATGGAACTTTTGTGAATAAGTAACATACACAATACCCAAGCTAGGTGATATGTGG
            *********************************************************
```

Supplemental Fig. 6 Alignment of allelic sequences used for genotyping of *Phvul.011F058500* between *Phaseolus vulgaris* v2.1 (Pvu2.1), C90 and C85 (*Zip_l*) and B11 (*Zip_h*).

Coloured regions at the ends of the alignment indicate the regions of primers used.

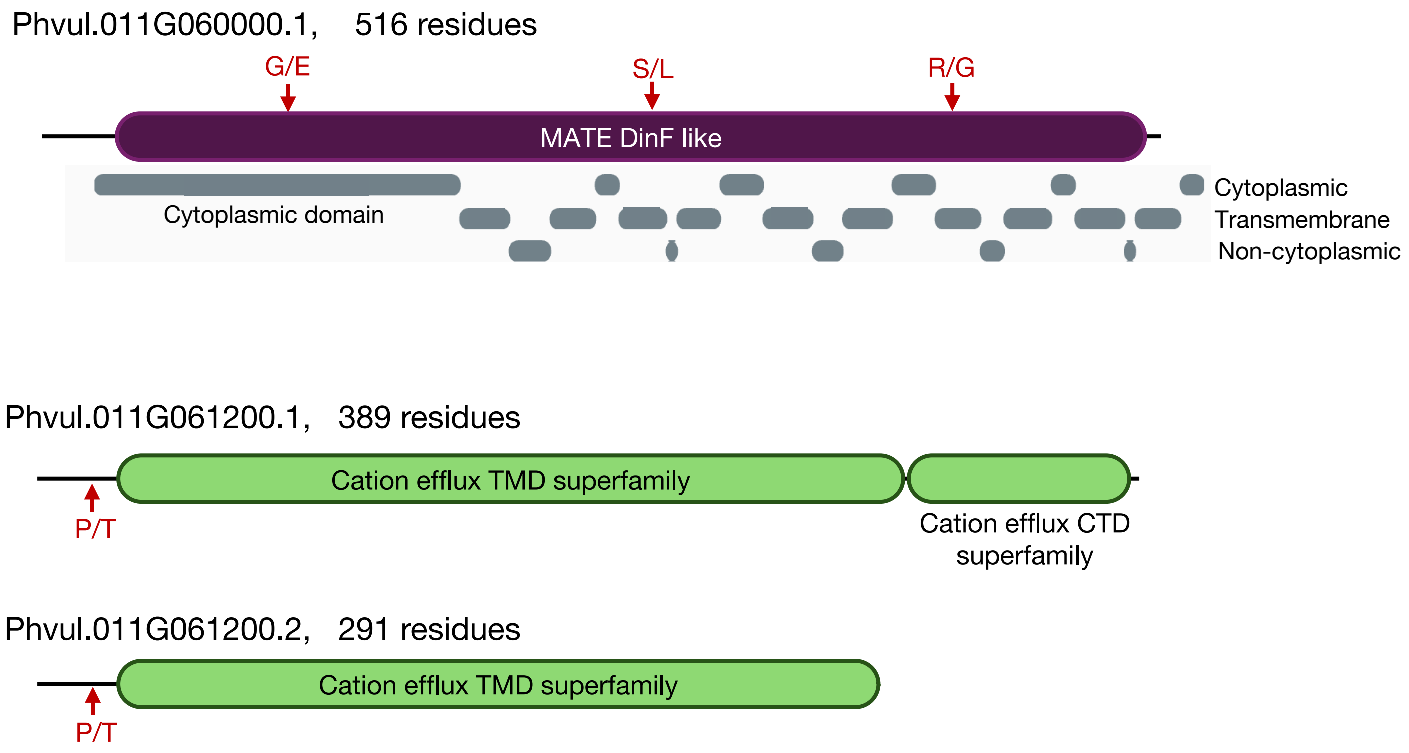

Supplemental Fig. 7 Predicted protein structures of the two candidate genes for ZN.

Predicted protein sequences encoded by the two candidate genes, *Phvul.011G060000* and *Phvul.011G061200*, are shown. Functional domains and conserved regions within the same protein family detected by Interpro are indicated as boxes. Positions at which differences in amino-acid residues between Pvu2.1 and C90 alleles were found are indicated by red arrows with one letter code of the residues (Pvu2.1/C90).

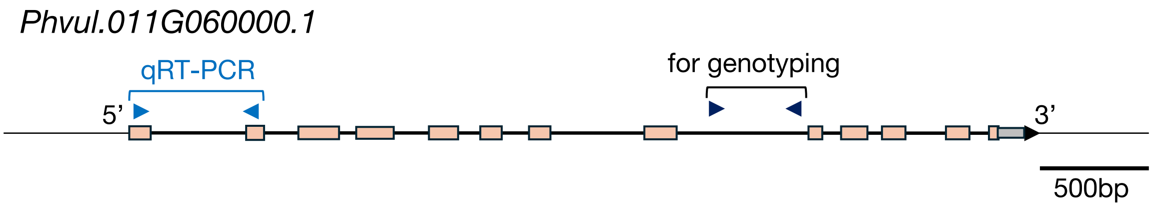

Supplemental Fig. 8 Gene structure of *Phvul.011G060000* and positions of the primers used in this study.

Pink boxes indicate coding regions, grey boxes indicate non-coding regions in the exons, and thick arrow indicates introns and direction of the transcription. Black short arrows indicate positions of the primers for genotyping. Blue arrow heads show the positions of primers used for qRT-PCR.

```
        Phvul.011g060000_GT_R  -->
C90     TACATGAAAAGTCACATTAGAGGTGATTTTCGACTGATTCAACATATAAGAGTTTTTACA
B18     TACATGAAAAGTCACATTAGAGGTGATTTTCGACTGATTCAACATATAAGAGTTTTTACA
Pvu2.1  TACATGAAAAGTCACATTAGAGGTGATTTTCGATTGATTCAACATATACAAGTTTTTACA
        ********************************* **************..**********

C90     CCGACCTAGCATAGCCATTAAACTCAATTTTGTTG-------AGCTCGACTAGAAATAAG
B18     CCAACCTAGCATAGCCATTAAACTCAATTTTGTTGGAGTTTCACATCCACTATAAATAAA
Pvu2.1  CCAACCTAGCATAGGCATTAAACTCAATTTGTTGGAGTTTTCCCCTTCACTAGAAATAAG
        **.*********** ***************  * *       . .*  **** ******.

C90     GACATTTCATTGTATAGAAATGAAT----------------------------------GC
B18     GACATTTCATAGTATAGAAGTGAAG----------------------------------GC
Pvu2.1  GACATTTTATAGTATAAAACTGAATGCAAAACTCACCTTATGAGTCATAAAACTGAATGC
        ******* **:*****.** ****                                  **

C90     AGATCTCACCTTATGAGTCAGTTTTATAAGGTTGAGTTAGACTTAAAATCCACTTCTTAA
B18     AGAATTCACCTCATGAACCGGTTTTATAAGGTTGAGTTAAACTTAAAGTCCACTTCATAA
Pvu2.1  AGAACTCACCTTATAAGTCGGTTATATAAGGTTAAGTTTGACTTAAAGTTCATTTCTTAA
        ***: ****** **.*. *.***:*********.****:.*******.* ** ***:***

C90     TATCAAAGCCCT--------------AACAAAAATCAGTTTAACTGATCATATTATATAA
B18     TATCAAAGCCCTTTAGA-ATCTATACTAAAAAAATTTGTTTAACTGATTATATTATATAA
Pvu2.1  TATCAAAATTATTTAAAGTTAATCATAACAAAAATTTGTTGAAGTGATCATAT-TTGTAA
        *******.  .*              :*.****** :*** ** **** **** :*.***
                                    <--  Phvul.011g060000_GT_R
C90     GTGGGTGCAATAATAATGATTGTTTTTTATATCGTGCCACTCTTTAGGAGCAAAAGA
B18     GTGGGTGCAATAATAATGATTGTTTTTTATATCGTGCCACTCTTTAGGAGCAAAAGA
Pvu2.1  GTGGGTGTAAGAATAATGATTGATTTTTATATCGTGCCACTCTTTAGGAGCAAAAGA
        ******* ** ***********:**********************************
```

Supplemental Fig. 9 Alignment of allelic sequences used for genotyping of *Phvul.011G060000* between *Phaseolus vulgaris* v2.1 (Pvu2.1, *MATE_h*), B18 (*MATE_m*) and C90 (*MATE_l*).
Coloured regions at the ends of the alignment indicate the regions of primers used.

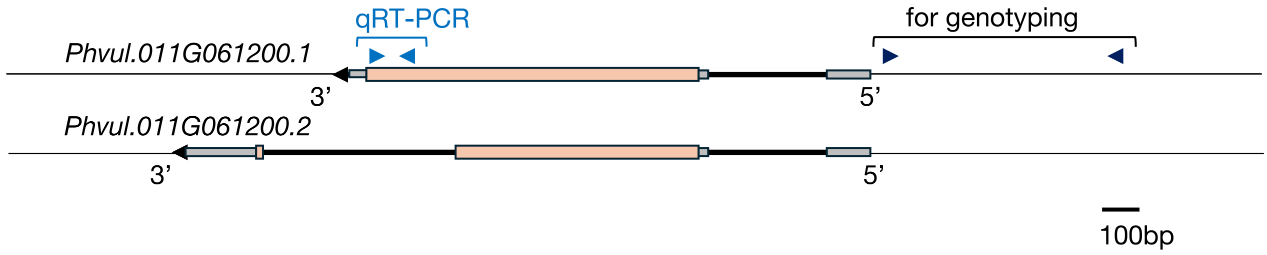

Supplemental Fig. 10 Gene structure of *Phvul.011G061200* and positions of the primers used in this study.

Pink boxes indicate coding regions, grey boxes indicate non-coding regions in the exons, thick arrows indicate introns and direction of the transcription. Black arrowheads show positions of the primers for genotyping. Blue arrowheads indicate primers used for qRT-PCR.

```
        MTP5up
Pvu2.1  GACCAAACTATACCACTACCATTCGATAACTTCTTTTATTTTCTAAAATAATTTCTACGT   60
C90     GACCAAACTATACCACTACCATTCGATAACTTCTTTTATTTTCTAAAATAATTTCTACGT   60
        ************************************************************

Pvu2.1  TTTTAATTTAATTGCTTCACCCAGAAAATGCTATTTACTTTTATTTATTTACCAATTTAA   120
C90     TTTTAATTTAATTGCTTCGCCCAGAAAATGCTATTTACTTTTATTTATTTACCAATTTAA   120
        ****************** *****************************************

Pvu2.1  AGCTAATCAATAATTTGGATTTCTCGTTTCTTATAATATAAAAAAAAAATCTGTTTTAGG   180
C90     AGCTAATCAATAATTTGGATTTCTCGTTTCTTATAATATAAAAAAAAAATCTGTTTTAGGA  180
        ************************************************     ***  *

Pvu2.1  AAAAAAAAAGTGCTTTACTTCCCTATCTTGAATTTTAAGATGATATAATGTCTTTTATTT   240
C90     AAAAAAAAAGTGCTTTACTTCCGTATCTTGAATTTTAAGATGATATATTTTATTTTGAAC   240
        ********************** ************************ * * ****

Pvu2.1  TGAATTGTAAGAAAATTTTAGTTTTAATACA--AGTGTACCAAACTATGAAGCTCAAACA   298
C90     TGATATTTTTATAAAGTATAACAATATTTTATAATTGTAAGAAAATTTTAGTTT------   294
        ***  * *    *** * **    ** *  *  * ****  *** * * *   *

Pvu2.1  CTCCTTTTAAATGGTGTGTCGGTGTTCGATGACACTCGTATAACACCTTTAGGACACGTA   358
C90     ----TAATACTTAGTGTAT-----------------------------------------   309
            *  **  * **** *

Pvu2.1  TATATATGAAGTGTCCAATTCAAAAAGTATTTGTTAGATTTCTAACAATTTTAATACGAT   418
C90     --------------CAAACTATGAAGCTCAACACTTCTTTTAAATGGTGTTGGCTTCGAT   355
                      * ** *   **  *      *   ***  *     **   * ****

Pvu2.1  TTTAACATAATTTTAAAAATAAAAAACACATTAATTTTCTAAAAAATCAAATTTTATTGT   478
C90     TGGAACACAATTTTAAAAATAAAAAATATATTAATTTTTAAAAAAATTAAATTTTATTGT   415
        *  **** ****************** * *********  ******* ************

Pvu2.1  AGAAATTGTTATTATGATTATAAAAACAAGAAACAAATCATAATGAACCGGTCATGAAAA   538
C90     ATTAATTGTTATTCTAATTATAAAAATAAGAAACAAATTATTGTGAACCAATCATGAAAA   475
        *  ********** * ********** *********** **  ******  *********

Pvu2.1  ACATCTTTCTATTTCAAAAAATAATTTTGAACATATTTGTGCACATAAATTTTTTATTGT   598
C90     ACATTTTTCTGTTTAAAAAAATAATTTGAAA-CA--TATTTATCACATAAATTTTTATTGT  532
        **** ***** *** *****  * **   * **  * * *       ** **********

Pvu2.1  CAATTTATATAATTCATAATTATATAATATATACATTCGTGTCCCCCTGTTCTACATTTT   658
C90     CAATTTATATAATTCATAATTATATAATATATAAATTCGTGTCTCGTAAATTTTATGAAT   592
        ********************************* ********* *     * *      *

Pvu2.1  AAAAATTTGATGTATTTTGGTGTTCGTGTAATATTAGTGTGTACGTGTCCTCTATTAGTA   718
C90     TTTGGATCGG-----TGTGGACTTCATGTA------------------------------   617
              * *      * ***  *** ****
                                                  MTP5dw
Pvu2.1  TATGTGTGATACCAACGTTTTCTATTGATAGTTTCACGCAACCAGAAGACATATAGATTG   778
C90     ----------CCAACGTTTCCTATTTGATAGTTTCACGCAACCAGAAGACATATAGATTG   667
                   * *   **     ************************************

Pvu2.1  AA   780
C90     AA   669
        **
```

Supplemental Fig. 11 Alignment of allelic sequences used for genotyping of *Phvul.011G061200* between *Phaseolus vulgaris* v2.1 (Pvu2.1, *MTP_h*) and C90 (*MTP_l*). Coloured regions at the ends of the alignment indicate the regions of primers used.
